# Bone morphogenetic protein 4 (BMP-4) and epidermal growth factor (EGF) inhibit metalloproteinase-9 (MMP-9) expression in cancer cells

**DOI:** 10.18632/oncoscience.144

**Published:** 2015-03-23

**Authors:** Nathalie Bibens Laulan, Yves St-Pierre

**Affiliations:** ^1^ INRS-Institut National de la Recherche Scientifique, INRS-Institut-Armand-Frappier, Boul. des Prairies, Laval, Québec, Canada

## Abstract

Matrix metalloproteinase-9 (MMP-9) plays a central role in the progression of the cancer. While a large number of studies have contributed to our understanding of the molecular mechanisms responsible for upregulating MMP-9 gene expression in normal and cancer cells, our knowledge on the signals that suppress MMP-9 expression is much more limited. Here, we report that EGF and BMP-4 cooperate to inhibit MMP-9 expression in cancer cells. Treatment with EGF reduces the expression of MMP-9 at both mRNA while augmenting BMP-4 expression. Interestingly, recombinant BMP-4 suppressed constitutive and PMA-induced MMP-9 expression in both fibrosarcoma and breast cancer cells. Addition of gremlin a natural inhibitor of BMP-4, inhibited the suppression of MMP-9 by EGF. The suppression of MMP-9 by BMP-4 likely occurs at the transcriptional level since BMP-4 suppressed MMP-9 mRNA expression and activation of a reporter vector encoding the human MMP-9 promoter. The suppressive effect of BMP-4 occurs via Smad1/5/8 and is specific since BMP-4 did not inhibit MMP-2 while BMP-2 was ineffective in suppressing MMP-9. Taken together, these results are consistent with a new paradigm for the role of EGF and BMPs in controlling MMP gene expression in cancer cells.

## INTRODUCTION

Tumor cell proliferation, invasion and metastasis are mediated, at least in part, through the degradation of the extracellular matrix by metalloproteinases (MMPs) locally produced by tumor and stromal cells. A point in case is MMP-9, which plays a significant role in cell invasion and metastasis in several cancers [1-4]. Consequently, a large number of studies have focused their attention on identifying and characterizing the molecular mechanisms that are responsible for increased expression of MMP-9 in cancer. In contrast, our knowledge on the molecular mechanisms that suppress MMP-9 remains largely unexplored and largely focused on epigenetic mechanisms, including DNA methylation [5-7]. Using an experimental mouse lymphoma model, where MMP-9 plays a central role, our group has recently shown that the EGF/EGR1 (epidermal growth factor/early growth response 1) pathway was involved in the repression of MMP-9 expression by stromal cells [8]. More specifically, we found that expression of MMP-9 in stromal was repressed by the activation of the *EGR1* gene induced by EGF secreted by tumor cells and it correlates with decreased MMP-9. How EGF/EGR-1 suppresses MMP-9, however, remains unknown.

Bone morphogenetic proteins (BMPs) were initially identified by Marshall Urist in 1960 [9]. Today, more than 30 members the BMP family have been reported. BMPs are extracellular proteins that bind membrane anchored serine/threonine receptors and induce a signal through intracellular R-Smads (1/5/8) signalization. Activated R-Smads form a heteromeric complex with the classic Smad4 which translocates to the nucleus and activates the transcription of BMP target genes [10]. Until recently, BMPs have been well known for their role in bone formation and as being powerful developmental regulators. The functions of BMPs, however, seems to go well beyond their role in bone formation. They also control a broad range of biological activities. During embryonic development, for example, BMP-4 is involved in gastrulation, mesoderm formation, hematopoiesis and development of several organs and tissues [11-13]. In fact, their role as critical signaling molecules that regulate cell fate decision, cell differentiation, cell survival and vasculogenesis, motility and cell adhesion are now well recognized. Not surprisingly, these properties have attracted the attention of an increasing number of investigators in the field of cancer. Abnormally high levels of BMPs have been reported in many cancer and have been associated with a poor prognosis, consistent with their ability promote dissemination, invasiveness and migration [14, 15]. Their role in cancer progression, however, remains ambiguous since several studies have shown that BMPs have anti-tumor functions [16, 17]. For example, while we know that BMPs can increase MMP expression in some cancer cells, such as gastric or prostate cancer cells [18, 19], others have shown that BMP-4 treatment of C3HT101/2 stem cells blocks MMP-3 and MMP-13 expression [20]. Such an ability to suppress MMPs has also been reported for BMP-4 and -6 [16, 21]. These results suggest the existence of a functional relationship between BMPs and MMPs. Here, we have investigated whether BMPs could be involved in the suppression of MMP-9 by EGF/EGR-1.

## RESULTS

### MMP-9 and BMP-4 expression in HT1080 cells following EGF/EGR-1 activation

**Figure 1:**
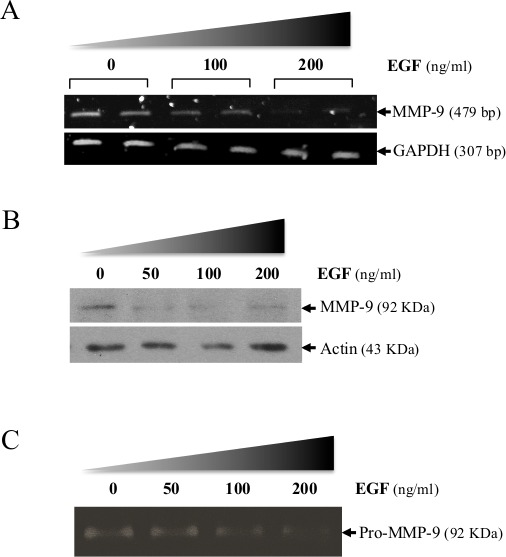
Suppression of MMP-9 in HT1080 cells following treatment with EGF (A) MMP-9 mRNA expression in absence or presence of recombinant EGF. Levels of transcripts were measured 16 h after adding EGF. GAPDH was used as loading and specificity control. Suppression of MMP-9 expression by increasing doses of EGF was confirmed at the protein level using (B) Western-blot analysis and (C) gelatin zymography. Data are representative of at least two independent experiments.

**Figure 2:**
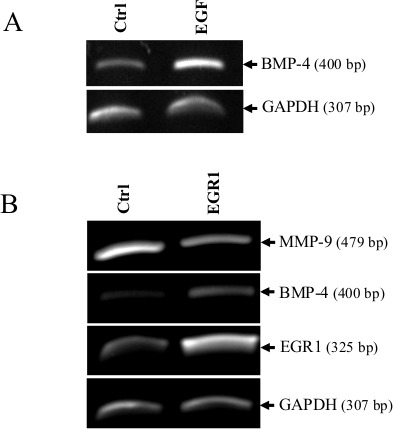
BMP-4 expression in HT1080 cells by EGF/EGR1 (A) BMP-4 mRNA expression in absence or presence of recombinant EGF. Levels of transcripts were measured 16 h after adding EGF. GAPDH was used as loading and specificity control. (B), BMP-4 and MMP-9 expression in HT1080 cells following stable expression of an expression vector encoding human EGR1. GAPDH was used as loading and specificity control. Data are representative of at least two independent experiments.

**Figure 3:**
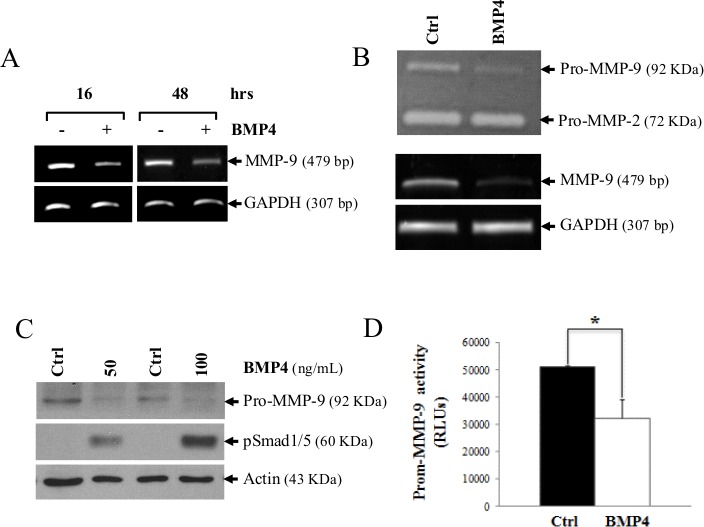
MMP-9 is decreased following BMP-4 stimulation in HT1080 cells (A) MMP-9 mRNA expression 24 and 48h following stimulation with recombinant BMP-4 (200 ng/ml). GAPDH was used as loading and specificity control. In (B), a zymogram (top gel) showing reduced MMP-9 secretion in supernatants of HT1080 cells treated for 16h with recombinant BMP-4 (200ng/ml). The lower panel shows the MMP-9 mRNA level of the treated cells. (C) Western blot analysis showing expression of MMP-9 and phosphorylation of Smad1/5 after treatment with human recombinant BMP-4. (D) Luciferase activity of in HT1080 cells transfected with a luciferase reporter vector containing the MMP-9-promoter following treatment with human recombinant BMP-4. Statistical analyses were carried out using Student's t test for unpaired samples. (* = *p ≤ 0,05;* ** = *p ≤ 0,005)*.

**Figure 4:**
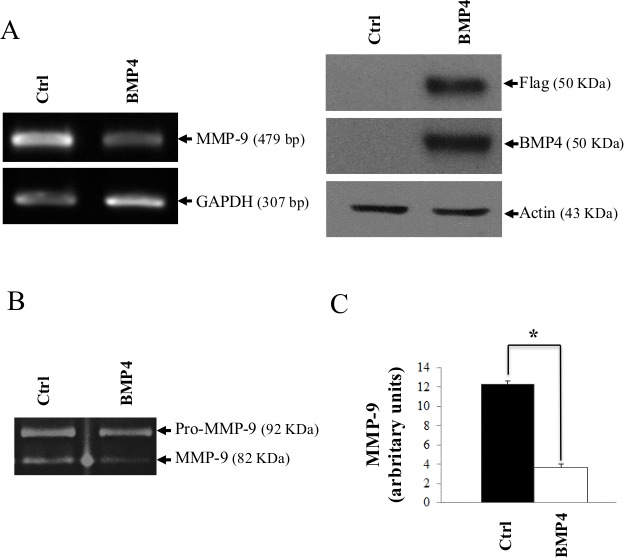
De novo expression of BMP-4 reduces MMP-9 gene expression (A) MMP-9 mRNA expression in mock-transfected HT1080 cells or HT1080 cells transfected with an expression vector encoding a flagged human BMP-4 (pCMV-BMP-4-Flag). GAPDH was used as loading and specificity control. The lower panel represents the control Western blot gels showing *de novo* expression of flagged BMP-4 in transfected cells. (B) Zymography (left panel) showing MMP-9 in the supernatant of transfected cells as in (A). (C) Quantitative analyses of MMP-9 expression by imaging densitometry is shown on the right histogram, which represents the means of independent experiments shown in (A). Data are representative of at least three independent experiments. (* = *p ≤ 0,05*)

**Figure 5:**
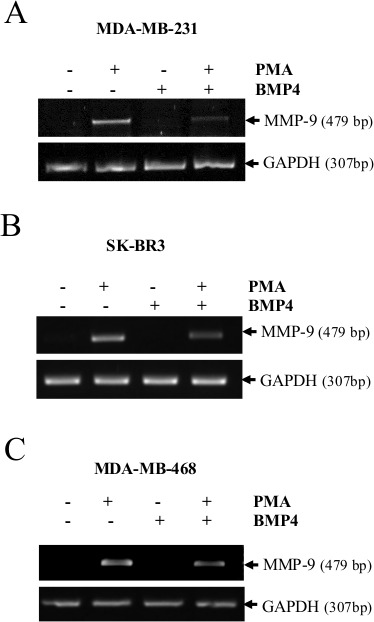
MMP-9 expression is suppressed by BMP-4 in MDA-MB-231, MDA-MB-468 and SKBR3 cells MMP-9 mRNA expression in PMA-stimulated cells (20ng/ml) treated with or without recombinant BMP-4 (50 or 100 ng/ml) in MDA-MB-231 cells (A), SK-BR3 (B) and MDA-MB-468 cells (C). GAPDH was used as loading and specificity control. Data are representative of at least three independent experiments.

Using an *in vivo* mouse model, we have previously shown that EGF suppresses MMP-9 gene expression [8]. Using the human HT1080 cells, an *in vitro* cell model commonly used to study the molecular mechanisms regulating human MMP-9, we have confirmed that EGF can suppress MMP-9 in a dose-dependent manner at the mRNA level (Fig. 1A). Similar results were obtained at the protein level as shown by Western blot and zymography (Fig. 1B-C). Such inhibition of MMP-9 by EGF was also observed in human breast cancer cell lines (Supplementary Fig.S1). Because BMP-4 has recently been shown to inhibit choroidal neovascularization by inhibiting VEGF and MMP-9 expression [21], we next investigated whether BMP-4 could be involved in the suppression of MMP-9 expression by EGF/EGR-1. We indeed found that suppression of MMP-9 by EGF in HT1080 cells correlated with increased *BMP-4* gene expression (Fig. 2A). Such increased in BMP-4 expression was also observed in stable transfectants of HT1080 cells expressing constitutive levels of EGR-1, a transcription factor known to be activated EGF (Fig. 2B). We thus tested whether treatment of HT1080 cells with recombinant BMP-4 (rBMP-4) could downregulate MMP-9 expression in HT1080 cells. Our results showed that rBMP-4 suppressed the expression of MMP-9 by HT1080 cells at both mRNA and protein levels (Fig. 3 A-B). The ability of BMP-4 to inhibit MMP-9 was specific since rBMP-4 did not modulate MMP-2. Moreover, no detectable decrease of MMP-9 was observed following treatment with recombinant BMP-2 (Supplementary Fig. S2). We also confirmed that rBMP-4 was biologically active, as shown by its ability to induce phosphorylation of Smad1/5/8 (Fig. 3C). The use of a luciferase reporter containing the human MMP-9 promoter further indicated that BMP-4 is likely to suppress MMP-9 expression at the transcriptional level (Fig. 3D). Ectopic expression of flagged-BMP-4 in HT1080 cells was also effective in suppressing MMP-9, further supporting the specificity of the inhibition (Fig. 4A-C). The ability of BMP-4 to inhibit MMP-9 expression was not restricted to HT1080 cells. We observed a similar decrease of MMP-9 by BMP-4 at both mRNA and proteins levels using the human breast epithelial MDA-MB-231, MDA-MB-468 and SK-BR3 cells, which express MMP-9 following stimulation with PMA (Fig. 5).

### Restoration of MMP-9 gene expression with gremlin

**Figure 6:**
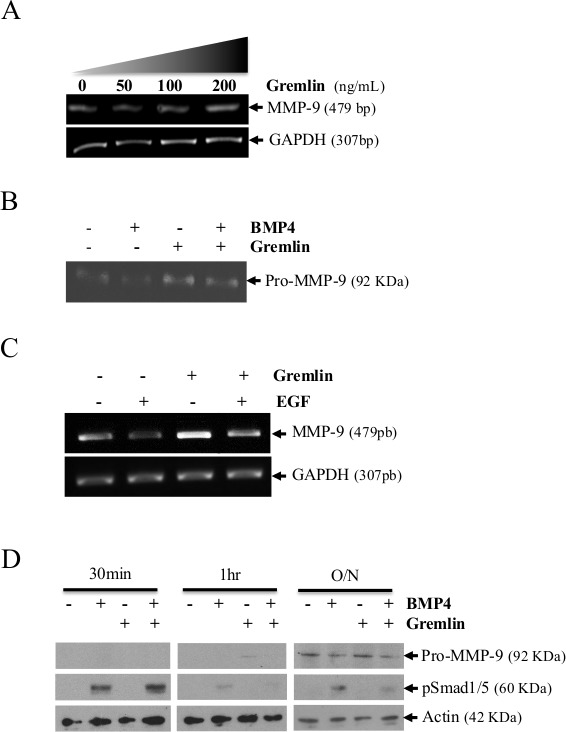
MMP-9 is increased following treatment of HT1080 cells with gremlin (A) MMP-9 mRNA expression in cells treated with increasing concentrations of gremlin. GAPDH was used as loading and specificity control. (B) Zymogram showing MMP-9 in the supernatant of HT1080 cells treated with recombinant BMP-4 in absence or presence of gremlin. (C) Kinetic analysis showing MMP-9 expression and phosphorylation of Smad1/5 in cells treated treatment with human recombinant BMP-4 with or without gremlin. Data are representative of at least three independent experiments. (D) MMP-9 mRNA expression in absence or presence of recombinant EGF and gremlin in HT1080 cells.

To further confirm that BMP-4 inhibits the expression of MMP-9, we used gremlin, a natural antagonist of BMP-4 which is highly expressed in cancer-associated stromal cells [22]. Our results showed that treatment of HT1080 cells with increasing concentrations of recombinant gremlin induced a detectable increase in MMP-9 expression in HT1080 cells at both mRNA and protein levels (Fig. 6A-B). Gremlin also blocked the EGF-mediated MMP-9 suppression (Fig. 6C). It also restored PMA-induced MMP-9 expression in cells treated with BMP-4. We also observed a significant increase in MMP-9 transcription and protein in MDA-MB-231 during treatment with gremlin (Supplementary Fig. S2). The effectiveness of gremlin to block BMP-4-induced signals was further confirmed by monitoring the phosphorylation of Smad1/5/8 (Fig. 6D).

### BMP-4 over-expression inhibited HT1080 cellular invasion

**Figure 7:**
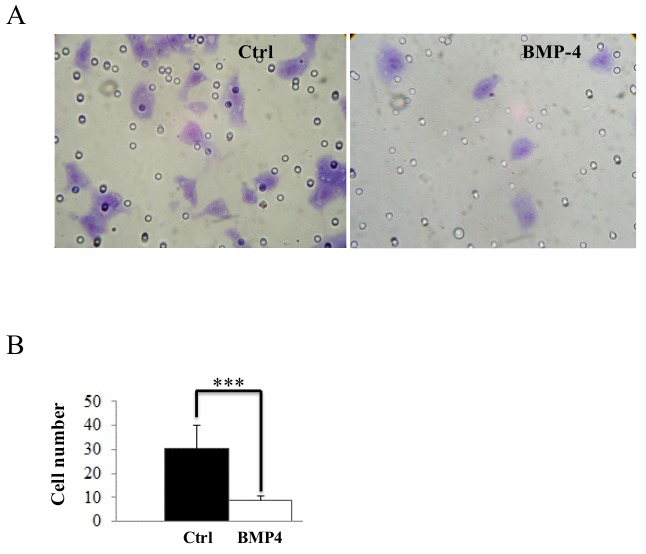
BMP-4 inhibits *in vitro* HT1080 cell invasion (A) HT1080 cell migration across Matrigel with or without recombinant BMP-4. (B) Migrated HT1080 cells were quantified. Data are average ± SEM; n = 10 unit areas. Data are representative of at least three independent experiments. (***= *p ≤ 0,001*).

Because elevated level of MMP-9 is well known to increase the migratory properties of tumor cells *in vivo* and *in vitro* [1, 2, 4], we next tested whether BMP-4 was able to suppress the invasion of HT1080 cells. For this purpose, we compared the *in vitro* transmigration of HT1080 cells through Matrigel in absence and presence of recombinant BMP-4. Our results showed that BMP-4 did indeed reduce the invasiveness of HT1080 cells as compared to control cells (Fig. 7).

### Smad6 affect the down-regulation of MMP-9 induced by BMP-4

**Figure 8:**
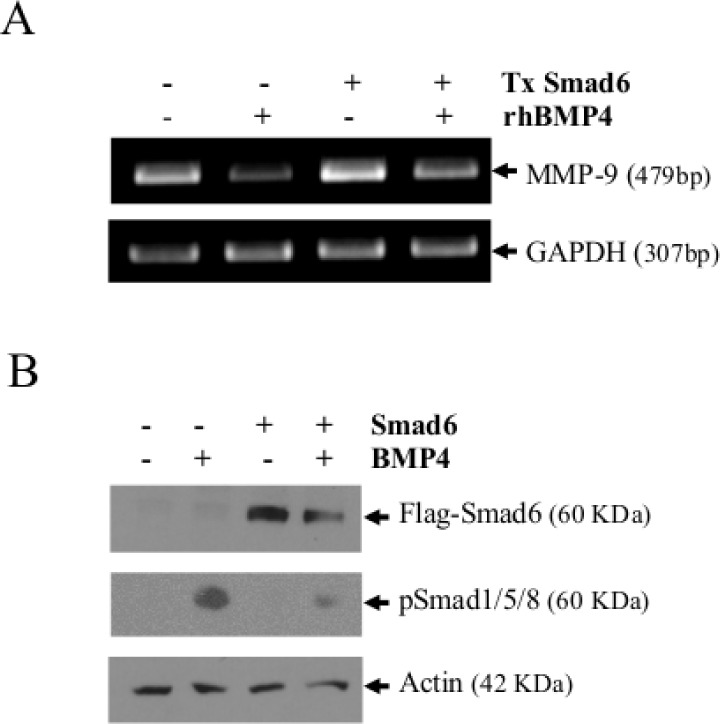
Smad6 reduces the ability of BMP-4 to suppress MMP-9 expression (A) MMP-9 mRNA expression in mock-transfected HT1080 cells or HT1080 cells transfected with an expression vector encoding a flagged Smad6 (pCS2-Smad6-Flag). MMP-9 expression was measured 16h after transfection. The lower panel represents the control Western blot gels showing *de novo* expression of Smad6 in transfected cells. GAPDH and actin were used as loading and specificity controls for RT-PCR and Western blot analyses respectively. In (B), a zymogram showing MMP-9 in the supernatant of HT1080 transfected cells. Data are representative of at least three independent experiments.

BMPs are well-known for their ability to induce signaling through the canonical pathway of Smad1/5/8, co-Smad (Smad4) and inhibitory Smad6 and 7. To determine whether this molecular pathway was involved in MMP-9 suppression by BMP-4, we carried a transient transfection of HT1080 cells using an expression vector encoding Smad6, which dimerizes with Smad1/5/8, thereby blocking its translocation to the nucleus [23]. We first confirmed that *de novo* expression of Smad6 was effective in blocking the phosphorylation of Smad1/5/8 while reducing the ability of BMP-4 to suppress MMP-9 (Fig. 8).

## DISCUSSION

A large number of studies have contributed to our understanding of the molecular mechanisms responsible for upregulating MMP-9 gene expression in normal and cancer cells. In contrast, our knowledge on the repressive signals that suppress MMP-9 expression remains fragmented. In a previous study, we had found that EGF suppressed the expression of MMP-9 *in vivo* and in HT1080 cells [8]. Here we have extended this work by showing that: 1) EGF-mediated suppression of MMP-9 was also observed in other cancer cells, most notably in human breast cancer cells; 2) stimulation with EGF also induced expression of BMP-4; this effect was observed in both HT1080 fibrosarcoma cells and MDA-MB-231, MDA-MB-468, SK-BR3 mammary epithelial cells; 3) the suppression of MMP-9 by BMP-4 likely occurs at the transcriptional level since BMP-4 suppresses MMP-9 mRNA expression and suppresses the activation of a reporter vector encoding the human MMP-9 promoter; BMP-4-mediated suppression of MMP-9 likely involves the Smad1/5/8 pathway. Moreover, transfection of a vector encoding Smad6 suggests that the suppressive effect of BMP-4 possibly involves the Smad1/5/8 pathway. Finally, we found that the effect of BMP-4 on MMP-9 was specific since BMP-4 did not inhibit MMP-2 while BMP-2 was ineffective in suppressing MMP-9.

BMPs are often associated with a poor prognosis in patients with different types of cancers. This seems to be largely due to the fact that BMPs target genes encoding osteoblast proteins and osteoblast-specific transcription factors [24]. For example, in prostate cancer, BMP-6 promotes osteoblastic activity of prostate cancer cells and confer them with a more invasive phenotype [25, 26]. Similarly, BMP-7 promotes expression of VEGF which contributes to the osteoblastic metastasis lesions [27]. A number of studies have shown, however, that BMPs may have a dual role in cancer and may in fact hamper tumor progression. For example, the bone morphogenetic protein pathway is active in human colon adenomas but inactivated in colorectal cancer [28]. A mutation of the receptor BMPR1A (bone morphogenetic protein receptor type IA) also promotes colon cancer [29]. Other studies have further shown that BMPs may also exert an anti-tumorigenic effect in breast cancer [16, 17]. In patients with familial adenomatous polyposis, BMP-2 can also block cell growth while promoting apoptosis of mature epithelial cells [30]. Such suppressive effect for BMPs seems to be particularly true for BMP-4. A recent clinical study has shown that patients high levels of BMP-4 have a better chance for survival when compared to patients with high-grade glioma who have a lower expression level of BMP-4 [31]. Also, BMP-4 negatively regulates tumor-initiating cells [32]. Whether such dual role in cancer is due to the ability to regulate MMPs is an interesting possibility since MMPs have also been reported to have a dual role in cancer. Our results herein and those from other groups showing that BMPs can suppress MMP gene expression supports this possibility. For example, Otto et al., have shown that BMP-4 blocks expression of MMP-3 and MMP-13 in C3HT101/2 stem cells [20]. BMP-6 has also been shown to inhibit MMP-9 expression in breast cancer cells [16]. Such suppression of MMP-9 by BMPs has also been reported during choroïdal neovascularization [21]. Our results showing that EGF induced BMP-4 further suggest the existence of a new functional relationship between both proteins that may be critical in the control of the invasive behavior of cancer cells. Taken together, these results are consistent with a new paradigm for the role of BMPs in controlling MMP gene expression in cancer cells. Future investigations will nevertheless be needed to solidly establish such a model and how the canonical Smad pathway is involved. While our preliminary studies with Smad6 are interesting in this regard, it is important to note that BMPs can activate the non-canonical MAPK (p38, MEK/ERK), Tak1/Tab1 and PI3K pathways [33, 34]. Interestingly, these non-canonical signal pathways are widely recognized to activate various transcription factors, including Nuclear factor-kappaB (NF-κB) [35], a transcription factor well-known for its ability to modulate MMP-9 expression [36]. Future investigations will thus be needed to identify which of these pathways are involved.

Overall, our study supports the idea that targeting EGF/BMPs could be a valuable alternative to inhibit MMP-dependent tumor progression, at least in some types of cancer. Cautions need to be taken, however, since MMP activity is not limited to extracellular matrix degradation and extends to proteolysis of various proteins bound to the cell membrane or even proteins secreted by cells. The repertoire of MMP substrates, or degradome, does indeed vary depending on the type and the different grades of tumors and in many cases, local increase of MMPs may have different impact on tumor progression [37]. Clearly, prudency will be needed to fully exploit the functional relationship between BMPs and MMPs for the treatment of cancer. Lessons from the failure of MMP inhibitors in clinical trials will be valuable to this regard.

## MATERIALS AND METHODS

### Cell lines and reagents

The human MDA-MB-231, MDA-MB-468, SKBR3, MCF7 and HT1080 cell lines were obtained from the American Tissue Culture Collection (ATCC, Manassas, VA). The MDA-MB-231 MDA-MB-468, MCF7 and HT1080 cells were maintained in culture in Dulbecco's modified Eagle complete medium (DMEM) [supplemented with 10% (v/v) FCS, 2 mmol/L L-glutamine and 10 mmol/L Hepes buffer]. The SKBR3 cell was maintained in culture in McCOy's medium [supplemented with 10% (v/v) FCS, 2 mmol/L L-glutamine and 10 mmol/L Hepes buffer]. Recombinant human EGF was purchased from Prospec Technogene (Ness Ziona, Israel). PMA (Phorbol-12-myristate-13-acetate) was purchased from Sigma-Aldrich (St. Louis, MI). Recombinant Human BMPs and Gremlin were obtained from Peprotech (Rocky Hill, NJ, USA). All other reagents were purchased from Sigma-Aldrich, unless otherwise indicated.

### RNA extraction and semi quantitative RT-PCR

Total RNA was isolated using Trizol reagent according to the manufacturer's instructions (Invitrogen, lifetechnologies, Burlington, ON, Canada). After reverse transcription, cDNAs were amplified using the following conditions: 94°C for 1 min, followed by 30-35 cycles of the following: 94°C for 1 min, 58-64°C for 1 min (depending on the primers), and 72°C for 1 min, followed by a final extension step at 72°C for 10 min. PCR was performed in a thermal cycler (MJ Research, Watertown, MA). PCR assays using equal amounts of RNAs that were reverse-transcribed and amplified by PCR for 25 to 40 cycles with gene-specific primers (Table S1) confirmed that the amplification was in the linear range for each gene. As an internal control, amplification of glyceraldehyde-3-phosphate dehydrogenase (GAPDH) mRNA was carried out by RT-PCR using specific primers. Amplified products were analyzed by electrophoresis on 1 % agarose gels using SYBR Safe DNA gel (Invitrogen) staining and UV illumination.

### Vectors, transfection and luciferase assays

The vector encoding Smad6 (pCS2-Flag-Smad6) was obtained from Addgene (Cambridge, MA). The vector encoding BMP-4 (pCMV-Flag-BMP4) was obtained from Sino Biological Inc. (Benjing, China). The plasmid encoding the luciferase reporter vector containing a fragment encompassing the essential consensus sequences for the transcriptional activity of the *mmp-9* promoter (pGL3-MMP-9) has been described [36]. For transfection, cells were plated at equal density 24h before transfection. Cells were then washed twice and transfected with 2 μg of DNA using DNAfectin 2100 according to manufacturer's protocol (ABM, Richmond, BC, Canada). After transfection, cells were incubated in complete medium at 37°C in 5% CO2 for 4 h. The culture medium was then changed to complete DNEM medium for 20 h. For zymography only, the culture medium was changed for serum free medium for 16 h. Luciferase activity was measured using the Luciferase Assay System protocol (Promega, Madison, WI, USA) and a luminometer (Lumat LB 9507, Berthold). The transfection efficiency was monitored by co-transfection with the pCMV/βGal plasmid encoding β-galactosidase (Promega, Madison, WI, USA). The β-galactosidase activity was detected by a colorimetric enzyme assay using α-nitrophenyl-β-galactopyranoside as a substrate. The ratio of luciferase to β-galactoside activity in each served as a measured of normalized luciferase activity.

### Gelatin Zymography

Zymography was performed in polyacrylamide gels that had been cast in the presence of gelatin as previously described [36]. Briefly, samples were suspended in loading buffer and, without prior denaturation, were run on a 7.5% SDS-polyacrylamide gel containing 0.5 mg/ml of gelatin. After electrophoresis, gels were washed to remove SDS and incubated for 18 h at 37°C in a denaturing buffer. Gels were subsequently stained with Coomassie brilliant blue G-250 and destained in 30% (v/v) methanol/10% acetic acid to detect gelatinase secretion. The proteolytic activity of MMP-9 was identified as a clear band on a blue background.

### Matrigel invasion assay

Invasion assay was carried out using Matrigel-coated invasion chambers (BD Biosciences). Briefly, cells were resuspended in culture medium without FBS at the concentration of 6 × 104 cells/ml and 500 μl of the cell suspension was seeded on the upper chamber. The lower chamber was filled with 700 μl of the culture medium without cells and 10% FBS was added as a chemoattractant. Cells were incubated with or without rhBMP4 for 18 h in a humidified tissue culture incubator, at 37°C, 5% CO2 atmosphere. After removing non-invasive cells with a cotton swab. Invasive cells adhering to membrane of the upper chamber were fixed with methanol 100% for 1 min and then stained with a Borax1% toluidine 1% solution for 1 min. Number of cells on the membrane was counted under a light microscope at 40X magnification.

## SUPPLEMENTARY MATERIAL FIGURES AND TABLE


