# Supplementary material for: Bone morphogenetic protein 4 (BMP-4) and epidermal growth factor (EGF) inhibit metalloproteinase-9 (MMP-9) expression in cancer cells
Source: Oncoscience. 2015 Mar 23;2(3):309–16. doi: 10.18632/oncoscience.144 (PMC4394136; doi:10.18632/oncoscience.144)
Supplement: Supplementary file 1 [file oncoscience-02-0309-s001.pdf]

Bone morphogenetic protein 4 (BMP-4) and epidermal growth factor (EGF) inhibit metalloproteinase-9 (MMP-9) expression in cancer cells

Supplementary Material

A

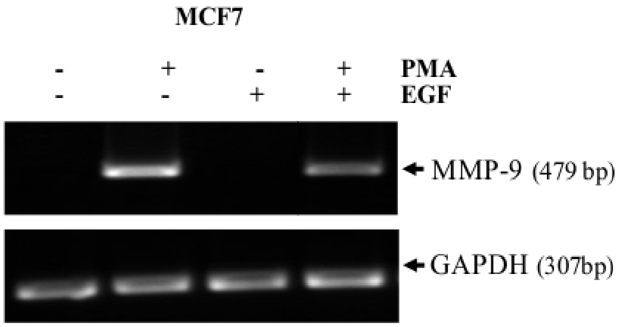

B

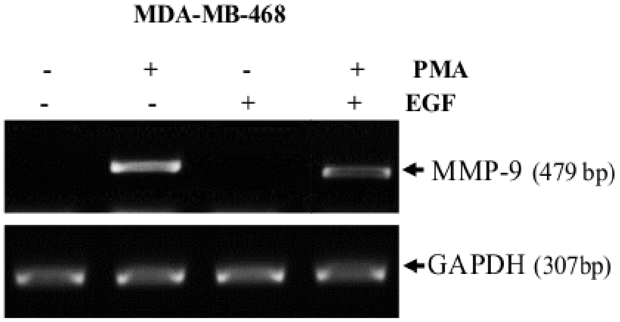

Figure S1: EGF suppresses MMP-9 expression in MCF7. MMP-9 mRNA expression in (A) MCF-7 and (B) MDA-MB-468 cells following treatment with human recombinant EGF. GAPDH was used as a loading control.

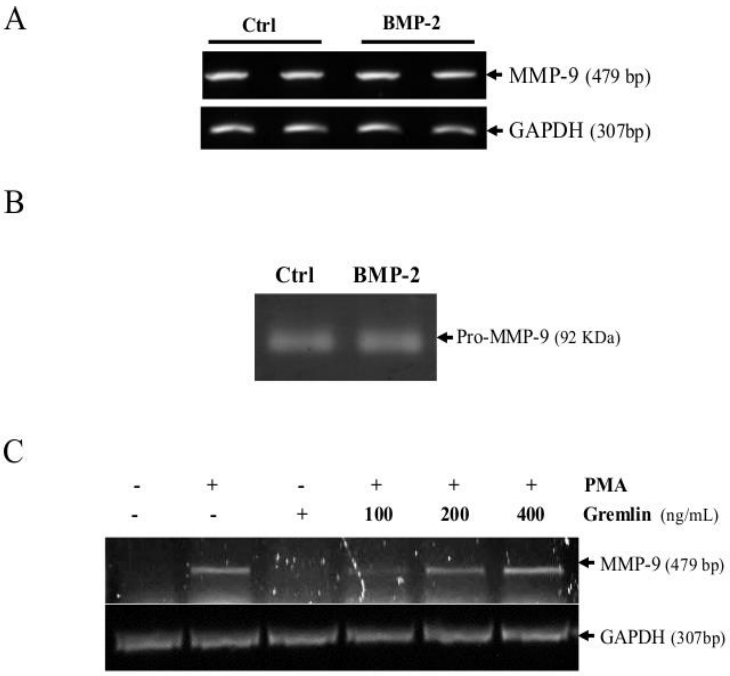

Figure S2: Effect of BMP-2 and gremlin on MMP-9 expression. MMP-9 expression at the mRNA (A) and protein (B) levels in HT1080 incubated following treatment with human recombinant BMP-2. GAPDH was used as a loading control. (C) PMA-induced MMP-9 mRNA expression in MDA-MB-231 cells following treatment with gremlin. GAPDH was used as a loading control.

Table S1: Gene-specific primers used for semi-quantitative RT-PCR

Human gene	Sense	Antisense
**MMP-9**	5’-caacatcacctattggatcc-3’	3’-cgggtgtagagtctctcgct-5’
**EGR1**	5’-tgggtgccgctgagtaaatg-3’	3’-ctgaccgcagagtcttttcctg-5’
**BMP4**	5’-ggcacagctatgttcatgggtcaga-3’	3’-ctttccccaggccaaggcacc-5’
**GAPDH**	5’-cggagtcaacggatttggtcgtat-3’	3’-agccttctccatggtggtgaagac-5’
